# Multi-Functional 3D-Printed Vat Photopolymerization Biomedical-Grade Resin Reinforced with Binary Nano Inclusions: The Effect of Cellulose Nanofibers and Antimicrobial Nanoparticle Agents

**DOI:** 10.3390/polym14091903

**Published:** 2022-05-06

**Authors:** Nectarios Vidakis, Markos Petousis, Nikolaos Michailidis, Vassilis Papadakis, Apostolos Korlos, Nikolaos Mountakis, Apostolos Argyros

**Affiliations:** 1 Mechanical Engineering Department, Hellenic Mediterranean University, Estavromenos, 71410 Heraklion, Greece; vidakis@hmu.gr (N.V.); mountakis@hmu.gr (N.M.); 2 Physical Metallurgy Laboratory, Mechanical Engineering Department, School of Engineering, Aristotle University of Thessaloniki, 54124 Thessaloniki, Greece; nmichail@auth.gr (N.M.); aposargy1@ee.duth.gr (A.A.); 3 Centre for Research & Development of Advanced Materials (CERDAM), Center for Interdisciplinary Research and Innovation, Balkan Centre, Building B’, 10th km Thessaloniki-Thermi Road, 57001 Thessaloniki, Greece; 4 Institute of Molecular Biology and Biotechnology, Foundation for Research and Technology—Hellas, 70013 Heraklion, Greece; vassilis_papadakis@imbb.forth.gr; 5 Department of Industrial Engineering and Management, International Hellenic University, 14th km Thessaloniki—N. Moudania, Thermi, 57001 Thessaloniki, Greece; apkorlos@ihu.gr

**Keywords:** nanocomposite, cellulose nanofiber (CNF), copper (Cu), copper oxide (Cu_2_O), antibacterial, vat photopolymerization, biomedical

## Abstract

This study introduced binary nanoparticle (NP) inclusions into a biomedical-grade photosensitive resin (Biomed Clear-BC). Multi-functional, three-dimensional (3D) printed objects were manufactured via the vat photopolymerization additive manufacturing (AM) technique. Cellulose nanofibers (CNFs) as one dimensional (1D) nanomaterial have been utilized for the mechanical reinforcement of the resin, while three different spherical NPs, namely copper NPs (nCu), copper oxide NPs (nCuO), and a commercial antimicrobial powder (nAP), endowed the antimicrobial character. The nanoparticle loading was kept constant at 1.0 wt.% to elucidate any synergistic effects as a function of the filler loading. Raman, thermogravimetric analysis (TGA), and differential scanning calorimetry (DSC) revealed the chemical/spectroscopic and thermal properties of the different manufactured samples. Scanning electron microscopy and Atomic Force Microscopy (AFM) revealed the morphology of the samples. Mechanical properties revealed the reinforcement mechanisms, namely that BC/CNF (1.0 wt.%) exhibited a 102% and 154% enhancement in strength and modulus, respectively, while BC/CNF(1.0 wt.%)/AP(1.0 wt.%) exhibited a 95% and 101% enhancement, as well as an antibacterial property, which was studied using a screening agar well diffusion method. This study opens the route towards novel, multi-functional materials for vat photopolymerization 3D printing biomedical applications, where mechanical reinforcement and antibacterial performance are typically required in the operational environment.

## 1. Introduction

The additive manufacturing (AM) family of technologies, in which three-dimensional (3D) printing is a subset, has been widely used in biotechnology, medical applications, aerospace, conductive devices, and sensors, among others [1,2,3,4,5,6,7]. Different 3D printing processes, such as material jetting [8], material extrusion (MEX) [9], powder bed fusion [10], and vat photopolymerization [11], operate with the same layer-by-layer procedure. In vat photopolymerization, liquid monomers are converted to solid polymers with an ultraviolet (UV) laser [12,13], achieving higher-resolution objects that are more accurate than MEX-built objects [14].

Compared to traditional manufacturing methods, 3D printing allows for more automation and fabrication of complicated geometries without molds or cutting tools [15,16,17,18,19], with each process being more suitable for specific industries [20,21,22]. Vat photopolymerization enables the possibility of manufacturing highly complex geometries without compromising repeatability [23,24,25,26]. The photopolymerization technique creates, compared to other AM technologies, a better formation, achieving a better fusion and bonding quality of layers [27,28]. However, applications of the vat photopolymerization technique are limited due to the low thermal stability and the brittleness of the manufactured parts [16,29,30,31].

The introduction of nanofillers into the polymer matrix to create 3D-printed nanocomposites with improved electrical, thermal, and mechanical properties is a recent advancement [32,33,34,35,36,37,38,39,40]. Fillers induce their properties in the nanocomposite materials, providing a multi-functional character to them [41,42,43]. For a long time, metals such as copper [44] and silver have been known to have wide antibacterial activity [45], with their performance imitating the key physiochemical characteristics of host defense peptides (HDPs) of the immune system [46]. The use of metal nanoparticles (NPs) is increasingly growing in the field over the last few decades [47]. Copper (Cu) has been extensively investigated for the improvement of the thermal conductivity of polymer composites with varied morphologies of particles, flakes, nanowires, and dendrites [48,49,50,51,52]. Metal oxide properties made them excellent candidates for several technological applications such as chemical sensors, fuel cells, optical devices, and solar cells, among others [53,54,55,56]. In this work, Cu_2_O is used as a nanofiller. Cellulose is a biopolymer made from the cell walls of trees and plants that has been extensively studied for use as a nanoscale fiber in nanocomposites in a variety of polymeric applications, including biotechnology for mechanical reinforcement [57]. Additionally, it has been used, in combination with metals exhibiting antibacterial performance such as silver [58], in medical applications, such as for wound infections requiring antibacterial performance [59]. Biological nanoparticles made from sustainable biomass, such as cellulose nanofibers (CNFs), are gaining popularity because of their high modulus, environmental benefits, low thermal expansion, and low cost [60], showing superior mechanical properties to glass and carbon fibers [61]. They are great alternatives to conventional fibers, providing enhanced mechanical properties, even at low loadings [62,63]. Polymers reinforced with CNFs nanoparticles or derivatives have attracted many scientists in recent decades [64,65,66].

In this work, for the first time, the effect of four different nanofillers suitable for biomedical applications are investigated in a biocompatible resin in vat photopolymerization 3D printing. More specifically Biomed Clear (BC) Resin (Product code: FLBMCL01) from Formlabs (Formlabs Ohio Inc., Millbury, OH, USA) was used as the matrix material, and three antimicrobial Nanopowder (NP) agents (Copper (Cu), Copper oxide (Cu_2_O), and the Antibacterial Powder “silver (Ag)-doped antibacterial” (AgDANP)) and a bio-friendly, ecological Nanopowder suitable and compatible with antibacterial applications (cellulose nanofibers (CNFs)) were used as fillers. With the help of a screening agar well diffusion method, the potential for antibacterial performance in the developed nanocomposites was investigated, leading to the development of multi-functional materials suitable for the vat photopolymerization process, taking advantage of the advantages of 3D-printing technology. Nanocomposites combining CNFs and each one of the three antimicrobial agents as fillers were also developed in this direction. The mechanical and thermal properties were experimentally assessed, and a morphological analysis was conducted for the evaluation of the specimens’ 3D-printing quality and the fractography mechanisms during testing. Pure resin and resin nanocomposites with CNF as a filler did not exhibit antibacterial performance as expected, while all the nanocomposites with antimicrobial agents as fillers showed a significant antibacterial response. All nanofillers tested induced significant improvement in the mechanical strength of the developed nanocomposites (more than 100% higher tensile strength for the BC/CNF nanocomposite and 65% higher tensile strength for the BC/Cu nanocomposite) compared to the matrix material. Only in the case of the nanocomposite combining both CNFs and Copper as fillers was the mechanical response reduced in the experiments conducted. These results show a strong potential for the development of multi-functional materials in vat photopolymerization 3D printing, with superior mechanical properties and antibacterial performance for medical applications. So far, no analogous research has been reported in the literature, according to the authors’ knowledge.

## 2. Materials and Methods

### 2.1. Materials

A medical-grade resin, with the trade name Biomed Clear (BC) Resin (Product code: FLBMCL01) from Formlabs (Formlabs Inc., Somerville, MA, USA), was used in this study as the photocurable vat photopolymerization resin and the matrix for the different nanocomposites. According to the safety data sheet (SDS) provided by the supplier, the composition of BC resin in weight percentage (wt.%) is ~50–70% of Bisphenol A dimethacrylate, 7–10% of 2-hydroxyethyl methacrylate monomer(s), 25–45% of Urethane dimethacrylate, and <2% photoinitiator(s). Biomed Clear (density: 1.09 g/cm^3^) has a viscosity of 1350 cps at 25 °C, while it is produced in compliance with the ISO 10993-1:2018, ISO 7405:2009/(R) 2015, ISO 18562-1: 2017 international standards, and according to EU Directive 2011/65/EU requirements. Biomed Clear is suitable for biocompatible applications requiring long-term skin or mucosal membrane contact. Parts printed with BioMed Clear Resin are compatible with common sterilization methods.

Cellulose nanofibers (CNFs) with a diameter of 10–20 nm and length of 2–3 μm, Cu NPs (purity: 99.95% metal basis, average size: 35 nm, surface area: 11–15 m^2^/g, bulk density 0.19 gr/cm^3^, spherical shape), CuO NPs (purity: 99.99% metal basis, average size: 38 nm, surface area: >20 m^2^/g, bulk density 0.8 gr/cm^3^, spherical shape), and Antibacterial Powder (AP) NPs (average size: 100 nm, surface area: 5 m^2^/g) were all purchased from Nanografi (Nanografi Nanotechnology, Ankara, Turkey). Procured CNFs have a length to diameter ratio (L/D) of approximately 150/1. According to the manufacturer’s technical datasheet, the cellulose crystallinity is 92% and decomposition temperature is 329 °C. CNFs are originally generated from wood-derived fibrils, with the raw material being cotton, while their surface is carboxymethylated, containing both hydroxyl and carboxyl groups. Antibacterial Powder (AP) has the commercial name “silver (Ag)-doped antibacterial” (AgDANP). These NPs are a rather low-cost mixture of metal oxides and other materials with antibacterial properties, containing, according to the manufacturer: Al_2_O_3_, HfO_2_, N_2_O, P_2_O_5_, TiO_2_, ZrO_2_, and Ag, having the following elemental analysis: P 16.15%, Zr 37.30%, Ag 4.00%, Y 0.55%, Sc 0.20%, and Al 0.14%. AgDANP was in nanopowder form with a particle size of 100 nm on average. According to the manufacturer’s datasheet, the bulk density is 0,39 g/cm^3^, its PH is 5.7, and the material can tolerate temperatures up to 350 °C, which is greater than the processing temperatures of extrusion and the 3D printing employed in this study.

### 2.2. Preparation of the Nanocomposite Resin Mixtures and Fabrication of the 3D-Printed Specimens

The BC resin nanocomposites were prepared by a shear mixing process [67]. The different nanocomposite formulations, namely (i) 1.0 wt.% of CNFs, (ii) 1.0 wt.% antimicrobial NP agent (Cu, CuO, and AP), and (iii) a binary mixture of 1.0 wt.% CNF:1.0 wt.% antimicrobial NP agent (in total: 2.0 wt.% filler loading), were prepared as follows: the photocurable resin has been weighed and then poured into the metallic container of the shear force mixer, and, as a next step, pre-weighed quantities of each of the nano additives were added in the resin under gentle shear mixing for 10 min. The whole formulation/mixture was then vigorously mixed, being exposed to 20.000 rpm for about 30 min at room temperature. At the end of each mixing process, the nanocomposite formulations were thoroughly degassed by being stored in a vacuum oven for 10 min to ensure that the air bubbles were removed before the layer-by-layer photopolymerization/3D-printing process. Specifically, the mixtures were placed in the 3D printer’s tank for the consecutive vat photopolymerization 3D-printing process. The 3D printer used was a Formlabs Form 2 (Formlabs Ohio Inc., Millbury, OH, USA) vat photopolymerization 3D printer with Formlabs Tank LT. The preparation of the necessary Gcode and the slicing process was implemented on the Preform 3.16 software tool. Biomed Clear resin was 3D printed with 100 μm layer height for every case tested. The specimens were 3D printed with a horizontal orientation on the 3D-printing table. After the 3D-printing process was completed, specimens were washed in a 99% pure isopropyl alcohol (IPA) solution for 15 min and dried for 5 min at room temperature and ambient conditions (this method was repeated twice), according to Formlabs specifications and guidelines. When the IPA cleaning process of the specimens was completed, a post-curing period of 60 min at 60 °C took place. Formlabs Form Wash and Form Cure (Formlabs Ohio Inc., Millbury, OH, USA) equipment has been used for the washing and the post-curing processes, respectively.

**Figure 1:**
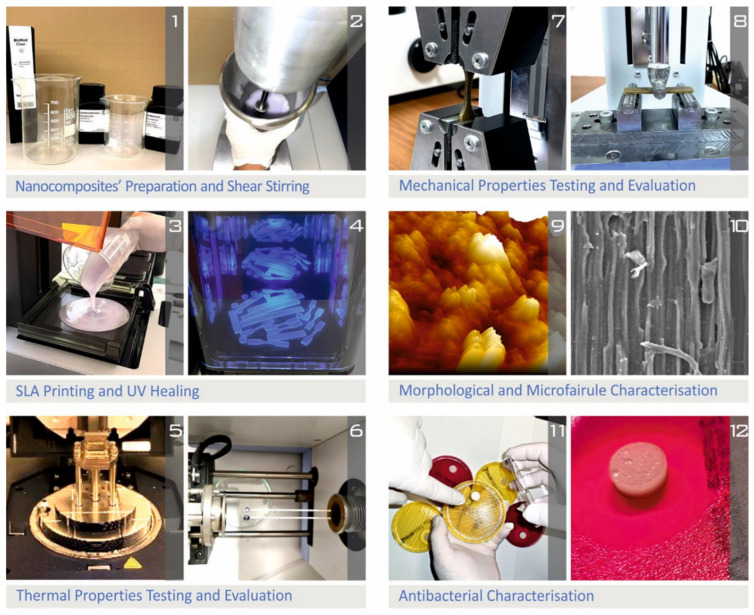
Flow chart illustrating the methodology followed to fabricate the different nanocomposites, as well as representative characterization techniques: (**1**) The additives used in this work, (**2**) materials mixing, (**3**) materials feeding in the 3D printer, (**4**) 3D printing process, (**5**) Mechanical testing (DMA), (**6**) Thermal properties (TGA), (**7**) Mechanical testing (tensile test), (**8**) Mechanical testing (flexural test), (**9**) morphological analysis (AFM), (**10**) morphological analysis (SEM), (**11**) (well agar diffusion), (**12**) antibacterial tests (inhibition zone).

**Figure 2:**
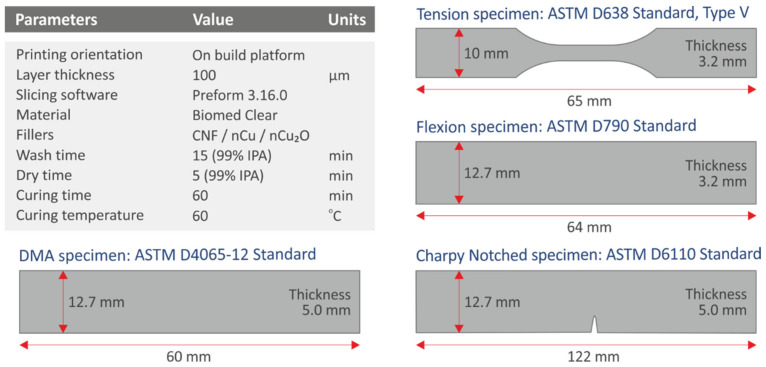
3D-printing and post-processing parameters of the vat photopolymerization AM method followed in this study. The different specimens’ geometry and dimensions are also depicted. For each mechanical test, samples were fabricated according to the requirements of the corresponding American Society for Testing and Materials (ASTM) international standard.

The flow chart in Figure 1 depicts the methods used to create the nanocomposites, as well as the manufacturing of the specimens and representative characterization methodologies for the 3D-printed samples. The complete list of vat photopolymerization 3D-printing settings utilized to create all the samples in the current study is shown in Figure 2.

### 2.3. Raman Spectroscopy and Thermal Analysis

Raman spectroscopy was conducted by a modified LabRAM HR Raman Spectrometer (HORIBA Scientific, Kyoto, Japan). A solid-state laser module at 532 nm central wavelength was used, which had a maximum laser output power of 90 mW, resulting in 40 mW of power on the sample. A 50× microscopic objective lens with a numerical aperture of 0.5 and a long working distance of 10.6 mm (LMPlanFL N, Olympus) delivered the excitation light and collected the Raman signals. The laser spot dimensions were approximately 1.7 μm of spot diameter and about 2 μm of axial focal length. The Raman spectral resolution was around 2 cm^−1^, with the 600 groves grating that was used. Each measurement was conducted with 10 s of acquisition time and five accumulations. The acquired Raman spectral range was set from 300 to 3100 cm^−1^, resulting in two optical windows or two acquisitions per point.

All the 3D-printed samples were subjected to thermogravimetric analysis (TGA) in an oxygen atmosphere. The measurements were carried out using a TGA/DTGA Perkin Elmer Diamond (Perkin Elmer, Waltham, United States) device at a constant heating rate of 10 °C/min over a temperature range of 40 °C to 550 °C. A Perkin Elmer Diamond (Waltham, MA, USA) apparatus was also used to perform differential scanning calorimetry (DSC), using a temperature cycle of 50 °C to 300 °C, with a 10 °C/min heating step, then cooling to 50 °C (a heat-cool-heat cycle is depicted for all samples).

### 2.4. Microstructural Investigations

Scanning electron microscopy (SEM) was used to examine the microstructure of the side surfaces of the 3D-printed specimens, as well as the fractured surfaces of the tensile specimens. SEM microstructural investigations were carried out using a JEOL JSM 6362LV (Jeol Ltd., Akishima, Tokyo, Japan) electron microscope at 20 kV acceleration voltage in high-vacuum mode. Samples were sputter-coated with Au coating to eliminate charging effects before the capturing of the images in the electron microscope. The electron microscope was also used for Energy-dispersive X-ray spectroscopy (EDS) analysis and for elements analysis in order to verify the elements in the developed nanocomposites. Specimens were not coated for the EDS analysis.

Atomic Force Microscopy in Tapping Mode (TM-AFM) was carried out in air at room temperature (23 °C) with a scanning probe microscope (MicroscopeSolver P47H Pro, NT-MDT, Moscow, Russia). Commercially available silicon cantilevers were employed, with a scanning frequency of 1 Hz, a tip cone angle of 20°, a cantilever spring constant of 35 N/m, and a tip radius of 10 μm. Measurements on impact specimens were used to characterize the surface roughness quality of the 3D-printed samples, and roughness values were determined using the Nova RC-1 NT-MDT image analysis software.

### 2.5. Mechanical Testing

Quasi-static tensile tests were performed following ASTM D638-02a. A type V specimen with a thickness of 3.2 mm was chosen following the standard, and a total of five (5) specimens were manufactured and tested for each case. The tensile tests were conducted utilizing an Imada MX2 (Imada Inc., Northbrook, IL, USA) tension/flexure test apparatus in tensile mode with standardized grips at a rate of 10 mm/min (in ambient conditions: 25 °C and 50% RH during the tests).

Flexural tests were also conducted on 3D-printed specimens (64.0 mm length, 12.4 mm width, and 3.2 mm thickness), following the ASTM D790-10 (three-point bending test with 52.0 mm support span). The same 3D-printing parameters were used for the manufacture of the flexural test specimens as they were for tensile test specimens. The three-point bending tests were performed using an Imada MX2 machine in flexural mode. The testing speed was also 10 mm/min, and a total of five (5) specimens were tested (in ambient conditions: 25 °C and 50% RH during the tests).

The ASTM D6110-04 was used to conduct Charpy’s impact testing on notched specimens. The dimensions of Charpy’s 3D-printed notched impact test specimens were as follows: 80.0 mm in length, 8.0 mm in width, and 10.0 mm in height (thickness). A Terco MT 220 machine for Charpy’s impact testing (Terco, Sweden) was used to test a total of five (5) specimens for each different material prepared in the work. For all the studies, the hammer’s release height was 367 mm and tests were conducted in ambient conditions: 25 °C and 50% RH.

Vickers microhardness measurements [68] were taken following ASTM E384-17. Before each set of measurements, the specimens’ surfaces were completely polished. The applied force was set to 100 gF, and the time of indentation was set to 10 s on an Innova Test 300-Vickers (Innovatest Europe BV, Maastricht, NL) apparatus. For all the prepared materials of the work, imprints were measured on five (5) separate specimens in ambient conditions: 25 °C and 50% RH.

A TA Instruments DMA850 instrument was used to perform Dynamic Mechanical Analysis (DMA) (Denmark). Samples were 3D printed with dimensions of 58.0–60.0 mm in length, 14.0–15.0 mm in width, and 2.7–3.2 mm in thickness. Before testing, samples were dried for at least 48 h at a temperature of 35 °C. In the DMA tests, a temperature ramp from ambient to 130 °C (and in some cases up to 135 °C) at a rate of 3 °C min^−1^ was used. The three-point bending fixture was used for testing. The samples were preloaded with 0.1 N. Throughout the experiments, α sinusoidal displacement with a constant amplitude of 30.0 μm and a frequency of 1.0 Hz was applied to the samples.

### 2.6. Biocidal Performance Investigation

The agar well diffusion method was used to investigate the antibacterial performance of the nanocomposites in this study [69] implemented for two separate bacteria, gram-negative *Escherichia Coli* (*E. coli*) and gram-positive *Staphylococcus aureus* (*S. aureus*), in a microbiological lab. For the tests, bacterium growth material (MC.2, C.010066 for *E. coli* and Chapman, C.010068 for *S. aureus*) suitable for each bacterium was utilized on Petri dishes that were 85 mm in diameter. The same parameters mentioned above were used to 3D print cylindrical specimens (12.4 in diameter and 3.2 mm in height) for all the materials used in this study (pure resin and nanocomposites). The bacteria were injected into the appropriate Petri dishes, and the specimens, one in each, were placed near the center of each Petri dish. The Petri plates were placed in an oven for 24 h at 37 °C to allow the antibacterial agent to diffuse into the material and impede the test bacteria’s germination and proliferation. The size of the developed inhibition zone on the Petri dishes was then measured with optical instruments.

## 3. Results and Discussion

### 3.1. Raman Spectroscopy and Thermal Analysis Results

**Figure 3:**
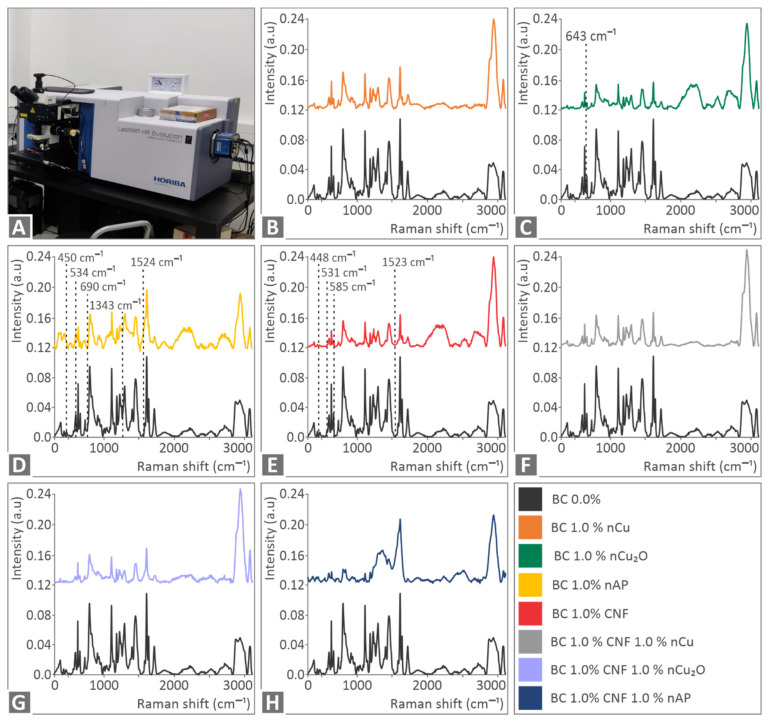
Raman Spectroscopy Analysis. In the first picture, the Raman Microscope used is depicted, followed by the related Raman Spectra from the different samples. Lastly, the color code for the different samples is described: (**A**) Raman spectra device, (**B**) spectra of BC 1.0 wt.% nCu vs BC, (**C**) spectra of BC 1.0 wt.% nCu_2_O vs BC, (**D**) spectra of BC 1.0 wt.% nAP vs BC, (**E**) spectra of BC 1.0 wt.% CNF vs BC, (**F**) spectra of BC 1.0 wt.% CNF 1.0 wt.% nCu vs BC, (**G**) spectra of BC 1.0 wt.% CNF 1.0 wt.% nCu_2_O vs BC, (**H**) spectra of BC 1.0 wt.% CNF 1.0 wt.% nAP vs BC.

Spectroscopy (RS) was performed to evaluate the materials at the molecular level. As depicted in Figure 3, the major contribution comes from the Biomed clear sample BC 0.0 wt.%. This is highly related (90% similarity) to the Raman spectrum for Epoxy. Sample BC 1.0 wt.% nCu shows no significant changes, except for some extra photoluminescence. Sample BC 1.0 wt.% nCu_2_O presents an extra Raman line at 643 cm^−1^ as expected. BC 1.0 wt.% nAP shows multiple changes in the Raman spectrum and at 450 cm^−1^ related to skeletal bending [70], 534 cm^−1^, 690 cm^−1^ related to low frequency vibrations of rings [71,72], 1343 cm^−1^ referring to C–C-H, C-O–H, and O-C-H [73], and 1524 cm^−1^ related to –C=C- vibrations [74]. BC 1.0% CNF shows some differences at 448 cm^−1^, 531 cm^−1^, 585 cm^−1^ related to Phenyl ring vibration [75], and 1523 cm^−1^. BC 1.0% CNF 1.0% nCu shows similarities, but with less significance with the samples of 1.0% nCu and 1.0% CNF, as expected. Similarly, BC 1.0% CNF 1.0% nCu_2_O shows the same changes with 1.0% CNF and 1.0% Cu_2_O. Lastly, BC 1.0% CNF 1.0% nAP presents similar Raman changes with 1.0% CNF and 1.0% nAP samples. Please see Table 1.

**Figure 4:**
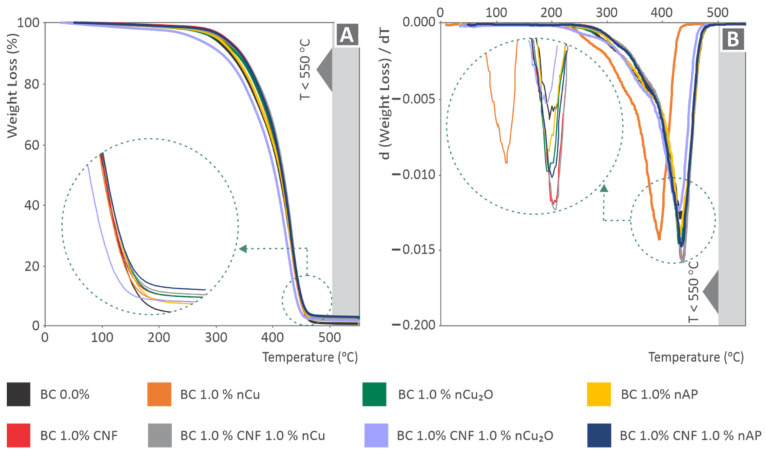
(**A**) TGA curves for all the materials investigated, with the *y*-axis being weight (percent) and the *x*-axis being temperature (°C). (**B**) DTGA curves for all the materials tested, with the *y*-axis being d(weight)/dT and the *x* axis being temperature (°C).

**Figure 5:**
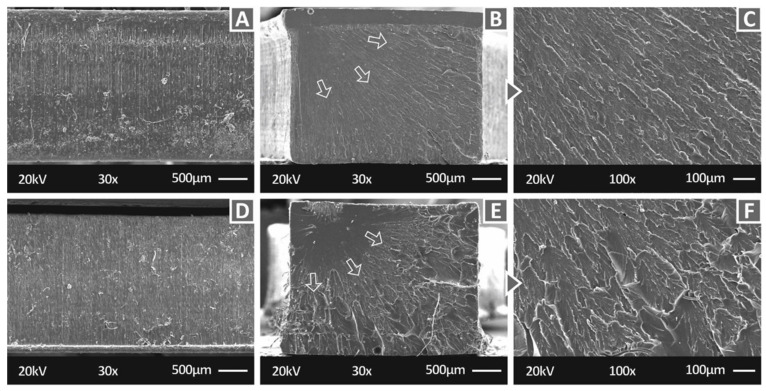
SEM images: (**A**) Pure Biomed Clear 30× magnification of the side surface, (**B**) Pure Biomed Clear 30× magnification of the fracture surface, (**C**) Pure Biomed Clear 100× magnification of the fracture surface, (**D**) Biomed Clear with 1 wt.% CNF 30× magnification of the side surface, (**E**) Biomed Clear with 1 wt.% CNF 30× magnification of the fracture surface, and (**F**) Biomed Clear with 1 wt.% CNF 100× magnification of the fracture surface. Arrows indicate the crack propagation on the fracture area for the two materials.

The produced Thermogravimetric Analysis (TGA)/Derivative Thermogravimetry Analysis (DTGA) graphs are shown in Figure 4. Figure 4A shows that, in all cases, the filler addition in the nanocomposites increases the degradation temperature of the nanocomposites slightly. The residues after full disintegration of the material, shown more clearly in the inset figure of Figure 4A, agree with the fillers’ ratio, confirming the assumption of a fine dispersion of the fillers in all produced nanocomposites. Figure 4B shows the DTGA results for all the materials that were evaluated. As it is shown, the maximum decomposition temperature remains constant in most cases. The nanocomposite with 1 wt.% Cu exhibits a different behavior, with the maximum decomposition rate temperature observed at a lower temperature than the other materials. Regarding the degradation rate, it increases for the 1 wt.% CNF and the 1 wt.% CNF and 1 wt.% Cu nanocomposites exhibit similar behavior as the pure material for the 1 wt.% Cu_2_O nanocomposite, while it decreases for the other nanocomposites when compared with the pure material, with the 1 wt.% CNF and 1 wt.% Cu_2_O nanocomposite having the lowest response among the materials studied in this work. These results show that the filler addition has a clear result of increasing the degradation temperature, while the effect of each filler regarding the maximum decomposition rate temperature and the degradation rate differs, indicating a different interaction between the filler and the matrix material in each case. All DSC curves had a similar tendency, showing that the 3D-printed samples have a cross-linked structure. Slight peaks in the graphs could be uncured resin residuals. In general, the addition of fillers to the Biomed Clear resin slightly reduced the absorbed energy, as can be observed in the graphs. The arrows in Figure 5 indicate a phase change in the material (Please see Figure S1, in the Supplementary Materials).

### 3.2. Microstructural Investigations

The morphological examination was performed on specimens of all filler loadings using Scanning Electron Microscopy (SEM) and Atomic Force Microscopy (AFM) to evaluate the fracture mechanism and the quality of the 3D-printing technique used in the current investigation. Additionally, the Electron Microscope was used for Energy-dispersive X-ray spectroscopy (EDS) to verify the elements in the materials prepared in this work.

Figure 5 shows the side of pure Biomed Clear (Figure 5A) and the crack surface of tensile specimens at two different magnifications (Figure 5B—30× and Figure 5C—100×). Figure 5 also shows the corresponding images for the Biomed Clear/CNF 1 wt.% nanocomposite (Figure 5D–F). The images from the side surface (Figure 5A,D) show a good 3D-printing quality, indicating that the 3D printing parameters were appropriate for the process and the addition of the filler did not affect the processability of the material. In the crack surfaces at 30× magnification, the crack propagation is visible in both materials. In both cases, the fracture started at a corner of the specimens and evolved on the surface, as indicated by the arrows in the Figure 5. In the pure material, the fracture started in a smaller area than in the CNF nanocomposite. In the higher magnification images, pure material (Figure 5C) exhibits a brittle failure, while the fracture surface of the CNF nanocomposite (Figure 5F) is significantly less brittle than the pure material, indicating that the introduction of the CNF NPs in this case impart the fracture with more ductile behavior. Additionally, a minor change in the cross-section shape of the tensile specimen can be observed on the upper side of the specimen in the lower magnification image (Figure 5B), which was caused during the vat photopolymerization 3D-printing process. This can be attributed to the high compression forces acting on the specimen during the formation of the initial layers, as the build platform tank gap is too small and the cross-sectional area in which the laser source cures resin is big enough. Such deformation is not of high importance as the total deformation is not exceeding 50 microns, which, according to the width of the specimen (3.2 mm), is a deformation of approximately 1.0%. It can be safely assumed that the recorded test results are not affected by such a minor abnormality in the specimen structure. It should be also mentioned that for the two materials presented in Figure 5, no EDS analysis was performed since no special elements were expected to be detected.

**Figure 6:**
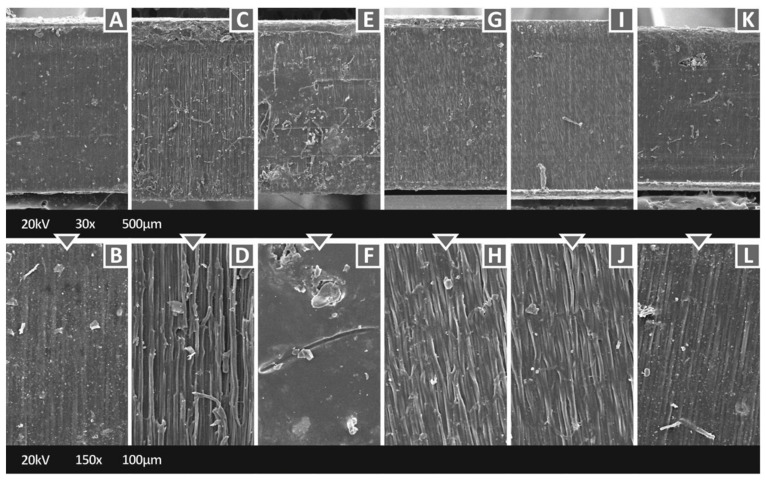
SEM images of the side surfaces of the specimens: (**A**) Biomed Clear 1 wt.% Antibacterial Powder, 30×, (**B**) Biomed Clear 1 wt.% Antibacterial Powder 150× side, (**C**) Biomed Clear 1 wt.% CNF and 1 wt.% Cu 30×, (**D**) Biomed Clear 1 wt.% CNF and 1 wt.% Cu 150×, (**E**) Biomed Clear 1 wt.% CNF and 1 wt. % Cu_2_O 30×, (**F**) Biomed Clear 1 wt.% CNF and 1 wt.% Cu_2_O 150×, (**G**) Biomed Clear 1 wt.% Cu_2_O 30×, (**H**) Biomed Clear 1 wt.% Cu_2_O 150×, (**I**) Biomed Clear 1 wt.% Cu 30×, (**J**) Biomed Clear 1% Cu 150×, (**K**) Biomed Clear 1 wt.% CNF and 1 wt.% Antibacterial Powder 30×, and (**L**) Biomed Clear 1 wt.% CNF and 1 wt.% Antibacterial Powder 150×.

Figure 6 shows the side surface of specimens manufactured with the nanocomposites of this work at two different magnifications (30× and 150×), except the case of the Biomed Clear with 1 wt.% CNF nanocomposite, which is depicted above. It is shown that the BC/AgDANP nanocomposite has a rather smooth side surface compared to the other nanocomposites. The roughest side surface is observed in the Biomed Clear 1 wt.% CNF and 1 wt.% Cu nanocomposites, while the other two nanocomposites with CNF and a second filler (Cu_2_O and Antibacterial Powder respectively) exhibit rather smooth side surfaces. The two nanocomposites with copper-based fillers (Copper and Copper oxide) also have rough side surfaces. Such behavior could be attributed to the 3D-printing parameters, which were the same for all materials and they were the ones of the pure resin.

**Figure 7:**
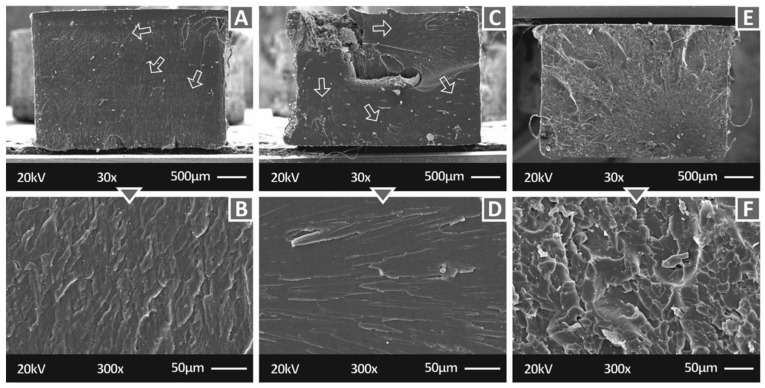
SEM images of the fracture surfaces of the specimens: (**A**) Biomed Clear 1 wt.% CNF and 1 wt.% Cu 30×, (**B**) Biomed Clear 1 wt.% CNF and 1 wt.% Cu 300×, (**C**) Biomed Clear 1 wt.% Cu_2_O 30×, (**D**) Biomed Clear 1 wt.% Cu_2_O 300×, (**E**) Biomed Clear 1 wt.% CNF and 1 wt.% Antibacterial Powder, 30×, and (**F**) Biomed Clear 1 wt.% CNF and 1 wt.% Antibacterial Powder, 300×. Arrows indicate the crack propagation on the fracture area for the two materials.

Figure 7 shows the fracture surface at two different magnifications (30× and 300×) of tensile specimens manufactured with three of the nanocomposites prepared in this work, i.e., Biomed Clear 1 wt.% CNF and 1 wt.% Cu 30×, Biomed Clear 1 wt.% Cu_2_O, and Biomed Clear 1 wt.% CNF and 1 wt.% Antibacterial Powder. While in all cases, the crack propagation is visible in the 30× magnification image, the fracture surfaces exhibit differences related to the brittleness of the fracture and the crack that initiated the fracture size, shape, and position in the fracture surface. In Figure 7, Biomed Clear 1 wt.% CNF and 1 wt.% Cu (Figure 7A,B), Biomed Clear 1 wt.% Cu_2_O (Figure 7C,D), and Biomed Clear 1 wt.% CNF and 1 wt.% Antibacterial Powder (Figure 7E,F) tensile test specimens fracture surfaces are depicted. The crack was initiated in all cases from a corner on the surface, while in the case of Biomed Clear 1 wt.% Cu_2_O, part of the specimen collapsed and was removed during the failure of the specimen. In this case, additionally, failure did not occur on one level, creating a step in the fracture surface. Biomed Clear 1 wt.% CNF and 1 wt.% Cu fracture surface exhibited a more brittle failure, while Biomed Clear 1 wt.% CNF and 1 wt.% Antibacterial Powder fracture surface was the more ductile one among these three. The shape of the cross-section is deformed in the cases of Biomed Clear 1 wt.% Cu_2_O and Biomed Clear 1 wt.% CNF and 1 wt.% Antibacterial Powder, while no deformation was observed in the case of Biomed Clear 1 wt.% CNF and 1 wt.% Cu. This deformation is explained above in the case of pure resin.

**Figure 8:**
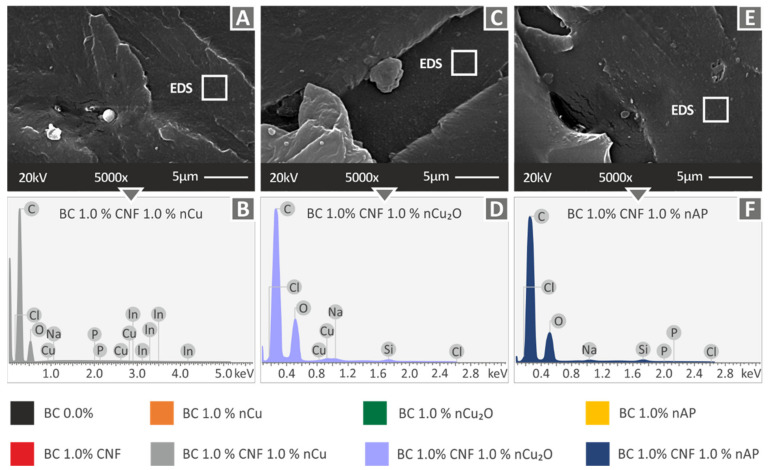
(**A**) Biomed Clear 1 wt.% CNF and 1 wt.% Cu SEM image with 5000× magnification of tensile test specimen’s fracture surface, (**B**) Biomed Clear 1 wt.% CNF and 1 wt.% Cu EDS graph, (**C**) Biomed Clear 1 wt.% CNF and 1 wt.% Cu_2_O SEM image with 5000× magnification of tensile test specimen’s fracture surface, (**D**) Biomed Clear 1 wt.% CNF and 1 wt.% Cu_2_O EDS graph, (**E**) Biomed Clear 1 wt.% CNF and 1 wt.% Antibacterial SEM image with 5000× magnification of tensile test specimen’s fracture surface, and (**F**) Biomed Clear 1 wt.% CNF and 1 wt.% Antibacterial EDS graph.

Higher magnification SEM images (5000×) of the fracture surface of tensile specimens of the three nanocomposites studied above, as well as their corresponding EDS graphs, which are shown in Figure 8. In all cases, no significant filler agglomerations were observed, indicating a good dispersion of the filler with the methodology followed in the work. At this magnification level, the failure mechanism can also be evaluated, and the observations of the lower magnification images were also verified at the higher magnification images. The EDS graphs verified the elements of the nanocomposites in all cases. In all cases, no high peaks for the additives’ elements were observed. This provides a qualitative indicator of good filler dispersion in the matrix material in the nanocomposites prepared in this study.

**Figure 9:**
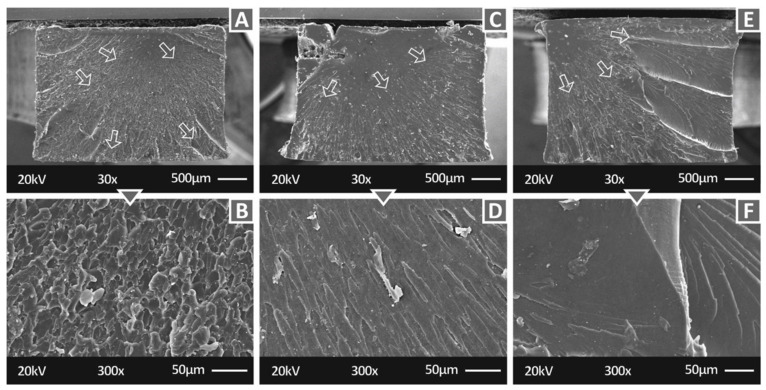
SEM images of the fracture surfaces of the specimens: (**A**) Biomed 1 wt.% Clear Antibacterial, 30×, (**B**) Biomed Clear Antibacterial wt.%, 300×, (**C**) Biomed Clear 1 wt.% Cu_2_O 30×, (**D**) Biomed Clear 1 wt.% Cu_2_O 300×, (**E**) Biomed Clear 1 wt.% Cu 30×, and (**F**) Biomed Clear 1 wt.% Cu 300×.

In Figure 9, Biomed 1 wt.% Clear Antibacterial (Figure 9A,B), Biomed Clear 1 wt.% Cu_2_O (Figure 9C,D), and Biomed Clear 1wt.% Cu (Figure 9E,F) tensile test specimens fracture surfaces are depicted. The crack was initiated for the first two cases (Biomed 1 wt.% Clear Antibacterial and Biomed Clear 1 wt.% Cu_2_O) in the middle of a side surface of the specimen, while in the third case (Biomed Clear 1 wt.% Cu), it was initiated on the corner of the surface, as before. More brittle behavior is observed in the case of Biomed Clear 1 wt.% Cu_2_O (Figure 9C,D), while in the other two materials the fracture surface shows deformation, indicating a more ductile failure, especially for the Biomed Clear 1 wt.% Cu (Figure 9E,F) material. The shape of the specimen’s cross-section is slightly deformed in these cases as well.

**Figure 10:**
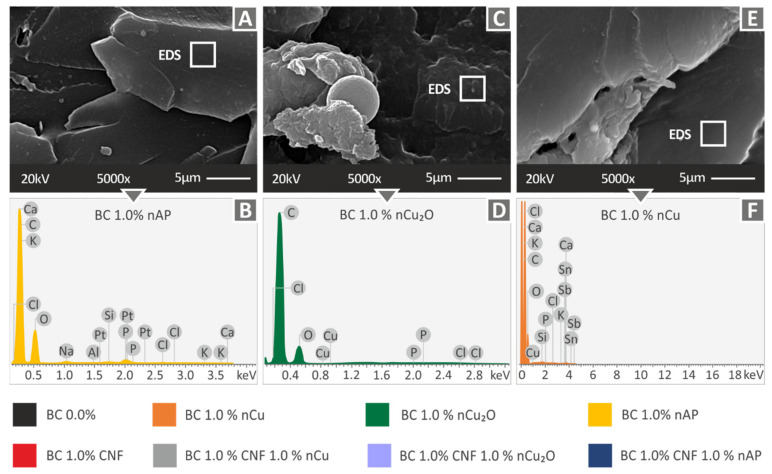
(**A**) Biomed Clear 1 wt.% Antibacterial SEM image with 5000× magnification of tensile test specimen’s fracture surface, (**B**) Biomed Clear 1 wt.% Antibacterial EDS graph, (**C**) Biomed Clear 1 wt. % Cu_2_O SEM image with 5000× magnification of tensile test specimen’s fracture surface, (**D**) Biomed Clear 1 wt. % Cu_2_O EDS graph, (**E**) Biomed Clear 1 wt.% Cu SEM image with 5000× magnification of tensile test specimen’s fracture surface, and (**F**) Biomed Clear 1 wt.% Cu EDS graph.

Figure 10 shows higher magnification SEM images (5000×) from the fracture surface of tensile specimens of Biomed 1 wt.% Clear Antibacterial (Figure 10A), Biomed Clear 1 wt.% Cu_2_O (Figure 10C), and Biomed Clear 1 wt.% Cu (Figure 10E) nanocomposites and the corresponding EDS graphs acquired for these materials. In these cases, no significant filler agglomerations were observed, except in the case of Biomed Clear 1 wt.% Cu_2_O (Figure 10C), indicating a good dispersion of the filler in the cases studied with the methodology followed in the work. The EDS graphs, as before, verified the elements of the nanocomposites in all cases. As with the rest of the nanocomposites analyzed with EDS, no high peaks for the additives’ elements were observed, which, as mentioned before, provides a qualitative indication of a good dispersion of the filler in the matrix material in the nanocomposites prepared in this work. Overall, analyzing the SEM images on the side and the fracture surface of the specimens, no clear trend can be derived for each filler or for the cases in which two fillers are combined and the overall filler loading in the nanocomposite is doubled. Zhang et al. [76] and Saba et al. [77] conducted similar research in vat photopolymerization 3D printing for wood composites and the results have a similar trend to the results of the current study regarding the brittleness of the materials with the addition of fillers and the effect of CNF in the matrix material in low concentrations.

Table 2 summarizes the AFM surface roughness measurements for all investigated materials. Lower surface roughness provides a qualitative indication of a fine dispersion of the filler in the matrix and possibly acts as a fine indicator of no agglomeration effects. Biomed Clear pure resin, Biomed Clear 1 wt.% Cu, Biomed Clear 1 wt.% CNF and 1 wt.% Cu, and Biomed Clear 1 wt.% CNF and 1 wt.% Cu_2_O exhibited low surface roughness values, with the remaining materials having higher values, but were within acceptable limits (Please see also Figure S2, in the Supplementary Materials).

### 3.3. Mechanical Testing Results

**Figure 11:**
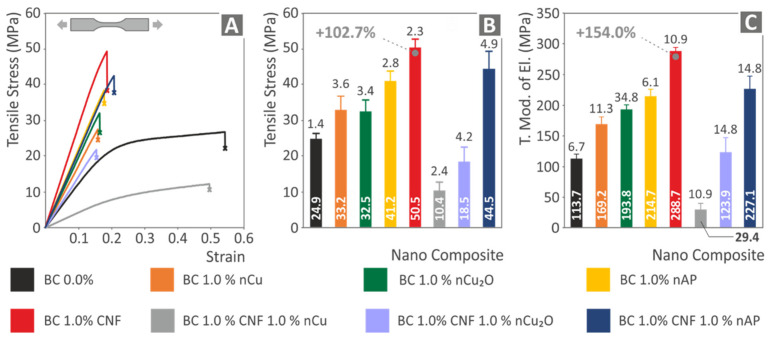
Tensile test results: (**A**) typical stress (MPa) vs. strain (mm/mm) graphs for all materials. (**B**) Average tensile strength (MPa) and deviation for all materials. (**C**) Average tensile modulus of elasticity and deviation for all materials.

The tensile test results are shown in Figure 11. The typical stress (MPa) to strain (mm/mm) curve for each of the current study’s evaluated materials is shown in Figure 11A. In comparison to pure Biomed Clear resin, all nanocomposite materials, except for the Biomed Clear 1 wt.% CNF and 1 wt.% Cu and Biomed Clear 1 wt.% CNF and 1 wt.% Cu_2_O nanocomposites, demonstrated a performance improvement, with the highest improvement observed in the Biomed Clear 1 wt.% CNF nanocomposite. This is an indication that the addition of copper-based fillers in nanocomposites with this specific matrix material and CNF as a filler degrades the mechanical response of the nanocomposite. The average tensile strength of the five specimens tested in each material prepared in this work, along with the calculated deviation is shown in Figure 11B. In the case of Biomed Clear CNF 1.0 wt.% nanocomposite, the average tensile strength (MPa) increased by 102.7% when compared to the pure matrix strength value, while the tensile strength was also improved for all other fillers and filler combinations, except the two cases mentioned above (Biomed Clear 1 wt.% CNF and 1 wt.% Cu and Biomed Clear 1 wt.% CNF and 1 wt.% Cu_2_O nanocomposites). In all cases, the calculated deviations were rather small, verifying the good quality of the process followed throughout the study. The corresponding average tensile modulus of elasticity and deviations are shown in Figure 11C. The tensile modulus of elasticity values follows the same trend as the tensile strength values, with the highest increase calculated also in the Biomed Clear 1 wt.% CNF (154.0% increase, when compared with the pure resin tensile modulus of elasticity). The Biomed Clear 1 wt.% CNF and 1 wt.% Cu nanocomposite exhibited the less stiff response among all the materials of the study, significantly lower than the pure resin, while the Biomed Clear 1 wt.% CNF and 1 wt.% Cu_2_O nanocomposite exhibited a similar and marginally stiffer response than the pure resin. Overall, the inferior performance of the Biomed Clear 1 wt.% CNF and 1 wt.% Cu nanocomposite agrees with the morphology examination, presented above, and can be attributed to the inferior 3D-printing quality of this specific nanocomposite with the 3D-printing settings used. Nanocomposite could probably not be completely cured and the dispersion of the fillers in the matrix was not completely achieved, resulting in an improper NPs network.

The results of this study agree with corresponding research from the literature. The addition of CNFs with 2.5 wt.% loading increased the mechanical strength of 3D-printed with Fused Filament Fabrication (FFF) Polylactic Acid (PLA) in the tensile tests by about 70% [78]. In another study, the addition of 1 wt.% CNF increased the tensile strength of 3D-printed PLA by about 100% [79] (102.7% in the current study, with different matrix material and 3D-printing process). Copper, as an additive, has a positive response in FFF 3D-printed PLA parts, with an increase of about 44% in the tensile tests [80] (about 32% in the current study, with different matrix material and 3D-printing process). No research was found in the literature for the matrix material of this study with these nanofillers, or with two different additives. Any differences can be attributed to the different matrix materials, the different grades of the additives, and the different 3D-printing processes employed in these studies. Still, the agreement of the results of this study to the literature verifies once more the reliability of the study’s results.

**Figure 12:**
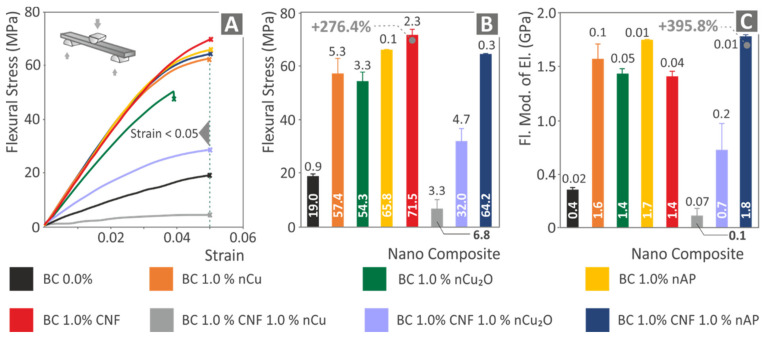
Flexural test results: (**A**) typical stress (MPa) vs. strain (mm/mm) graphs for all materials, (**B**) average flexural strength (MPa) and deviation for all materials, and (**C**) average flexural modulus of elasticity and deviation for all materials.

The flexural test results are depicted in Figure 12. The same trend with the tensile tests can be observed, with the nanocomposites exhibiting an enormous improvement in their performance in most cases. Figure 12A shows typical stress-strain graphs from the flexural tests for all materials tested. Experiments, as can be seen in the graphs, were terminated at 5% strain, following the ASTM D790 international standard instructions. Again, the Biomed Clear 1 wt.% CNF and 1 wt.% Cu nanocomposite exhibited an inferior performance compared with the pure resin, with all the other nanocomposites having an improved response in the tests. In agreement with the tensile tests, the Biomed Clear 1 wt.% CNF nanocomposite has the most improved performance, with a 276.4% higher flexural strength than the pure resin (Figure 12B). The average flexural modulus of elasticity and deviations are shown in Figure 12C. The flexural modulus of elasticity values follows the same trend as the flexural strength values, with the highest increase calculated in the Biomed Clear 1 wt.% CNF and 1 wt.% Antibacterial Powder nanocomposite, with an enormous 395.8% increase when compared with the pure resin flexural modulus of elasticity. Overall, the addition of the fillers of this work, in all cases studied except one (Biomed Clear 1 wt.% CNF and 1 wt.% Cu nanocomposite), have a positive effect on the flexural response of the materials.

Tensile toughness for all materials of this work, which is the absorbed energy during tensile testing and is calculated as an integral of the tensile stress to strain curve, is presented in Table 3. Tensile toughness does not follow the same trend as the tensile strength, with the pure resin absorbing more energy during the tensile test when compared with all the nanocomposites of this work. As such, the addition of the fillers in this work reduces the absorbed energy during the tensile tests. This is attributed to the reduced strain of the nanocomposites during the tests compared to the pure resin, which ultimately reduces the absorbed energy during the tests. Table 3 shows the calculated average Impact Toughness values and their deviations for all the materials prepared and tested in this work. In this case, some fillers improve the impact toughness of the matrix material (Biomed Clear 1 wt.% Cu, Biomed Clear 1 wt.% CNF, and Biomed Clear 1 wt.% Antibacterial Powder), while others showed inferior performance in this test (Biomed Clear 1 wt.% Cu_2_O, Biomed Clear 1 wt.% CNF with Cu, Cu_2_O, and Antibacterial Powder). The only clear trend, in this case, is that nanocomposites with two fillers exhibited inferior performance in this test when compared with the pure resin, with agrees with the remaining mechanical results, in which in most cases nanocomposites with two fillers were had lower performance than the nanocomposites with one additive. Biomed Clear 1 wt.% CNF nanocomposite exhibited superior performance in most of the mechanical tests, while the addition of a second copper-based filler in this material drastically reduced its response in the tests. Although the dispersion, according to the results and the investigation during the study is assumed to be fine in most cases, in the case of the nanocomposites with two fillers, the NPs network seems to be affected, with the addition of copper-based fillers, and the interaction between the fillers negatively affects the performance of the material in most cases. In addition, nanocomposites with two fillers, due to their higher loading, could probably reach a saturation level, which negatively affects their performance, when compared with the nanocomposites having one only of these fillers as additive. Table 3 presents the average Vickers Microhardness results and their deviations for all materials tested. Hardness at the microscale level was increased with the addition of the fillers in the matrix material in all the cases studied in the work. As such, the addition of these fillers hardened the material, and in this case, a different trend compared to other tests was observed. Biomed Clear 1 wt.% CNF with Cu nanocomposite showed the highest Vickers Microhardness values among the materials tested and in general nanocomposites with two fillers exhibited higher values among the tested materials and the addition of copper-based fillers leads to increased hardening of the material (Please see also Figure S3, in the Supplementary Materials).

**Figure 13:**
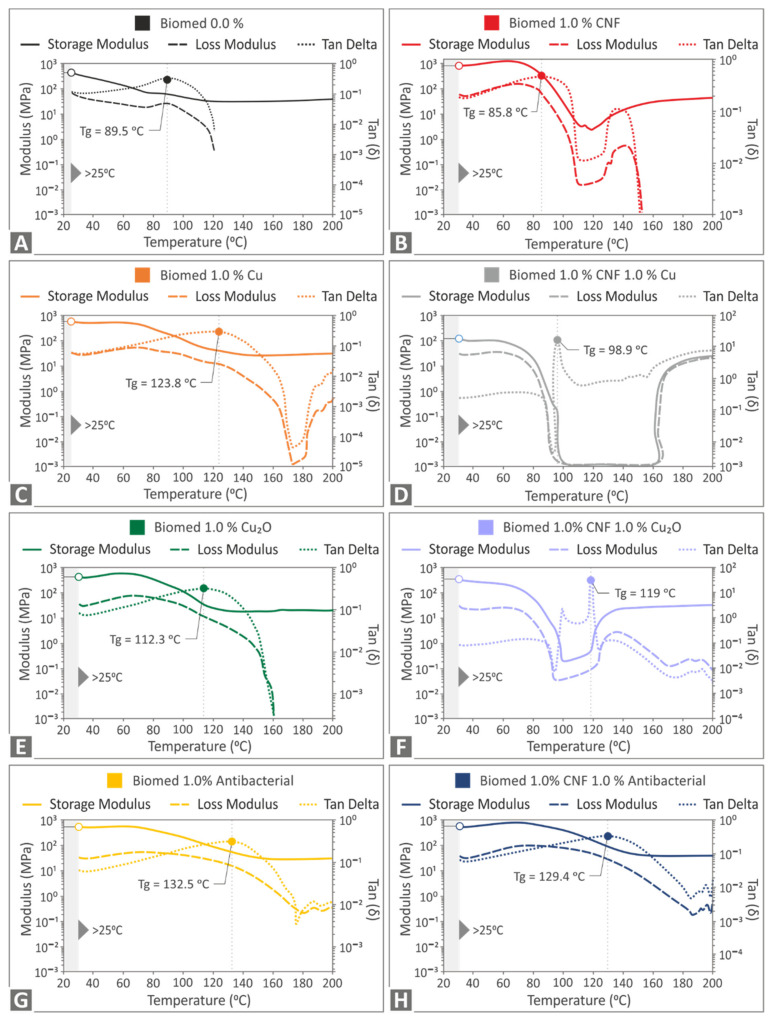
DMA curves: (**A**) Pure Biomed Clear, (**B**) Biomed Clear 1 wt.% CNF, (**C**) Biomed Clear 1 wt.% Cu, (**D**) Biomed Clear 1 wt.% CNF and 1 wt.% Cu, (**E**) Biomed Clear 1 wt.% Cu_2_O, (**F**) Biomed Clear 1 wt.% CNF and 1 wt.% Cu_2_O, (**G**) Biomed Clear 1 wt.% Antibacterial Powder, and (**H**) Biomed Clear 1 wt.% CNF and 1 wt.% Antibacterial Powder.

The findings of the DMA investigation are presented in Figure 13. Storage modulus (MPa), loss modulus (MPa) -left axis-, and tan (delta) -right axis- findings are shown for all materials tested. After computing the highest tan (delta) at the corresponding temperature for all of the tested materials, the glass transition temperature (Tg) is presented in the graphs. When compared to pure Biomed Clear resin, the storage modulus, and the tan (delta) of the nanocomposites vary. Loss modulus generally increases when compared to pure resin. Glass transition is also affected with the addition of the filler, and it is increased (Biomed Clear 1 wt.% CNF and 1 wt.% Cu nanocomposite, Figure 13D) and significantly increased in specific nanocomposites (Figure 13C,E–H), while it decreases in only one case (Biomed Clear 1 wt.% CNF nanocomposite, Figure 13B) compared to the pure resin. In the nanocomposites with CNFs, the viscoelastic behavior changes during the tests, as can be observed in the corresponding graphs.

### 3.4. Biocidal Performance Results

**Figure 14:**
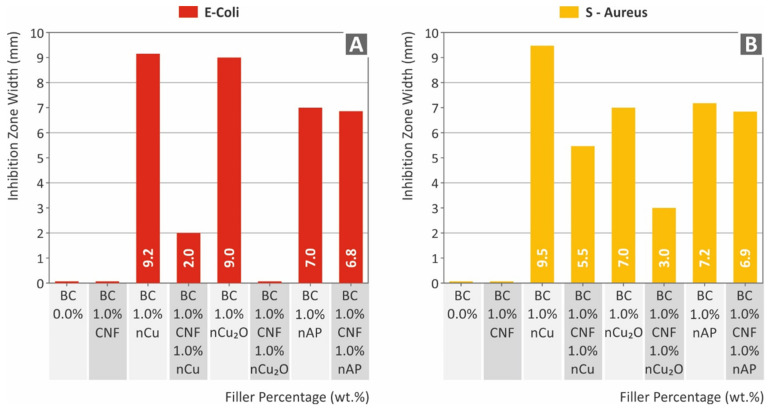
Comparison of the inhibition zone measurements for each material examined: (**A**) gram-negative *E. coli*; (**B**) gram-positive *S. Aureus*.

The antibacterial performance findings for the two bacteria tested are presented in Figure 14 below. As expected, pure resin and Biomed Clear 1 wt.% CNF does not exhibit antibacterial performance. Additionally, the Biomed Clear 1 wt.% CNF with 1 wt.% Cu_2_O did not show any antibacterial performance against gram-negative *E. coli*. As with the *E. coli* results, pure resin and Biomed Clear 1 wt.% CNF does not exhibit antibacterial performance, while the Biomed Clear 1 wt.% CNF with 1 wt.% Cu_2_O showed antibacterial performance against gram-positive *S. Aureus*. As is shown in Figure 14, there are significant differences between the antibacterial performance of the fillers, which additionally have a different response for each bacterium studied, although the trend is the same for the two bacteria. The copper-based fillers developed wider IZs, showing a very good antibacterial response in the screening process followed in the work. Pure copper showed an improved performance when compared to the copper oxide nanofiller and both copper-based fillers performed better in the gram-negative *E. coli* than the gram-positive *S. Aureus*. This is expected since the overall antibacterial mechanism of copper and its oxides are not yet positively determined in literature [44]. On the other hand, when copper-based fillers were mixed with CNFs, the nanocomposites had significantly inferior performance, which is an indication of an insufficient interaction between the two nanofillers in these cases. The Silver Doped Antibacterial Powder showed sufficient antibacterial performance in the work for both bacteria, developing IZs with similar widths in both cases.

## 4. Conclusions

Biomed Clear, a medical-grade UV-cured resin for vat photopolymerization 3D printing, and four different nano additives used in applications requiring antibacterial performance were employed for the fabrication of nanocomposites suitable for vat photopolymerization 3D printing, using common laboratory equipment. One additive was introduced in the matrix material for each nanocomposite, while combinations with two nanofillers added to the polymer matrix were also investigated. The aim was to evaluate the effect of each filler on the antibacterial and the mechanical performance of the matrix material, towards the cost-effective development of multi-functional nanocomposites exploiting the advantages of vat photopolymerization 3D printing and depicting enhanced performance for medical and other applications requiring such specifications.

**Figure 15:**
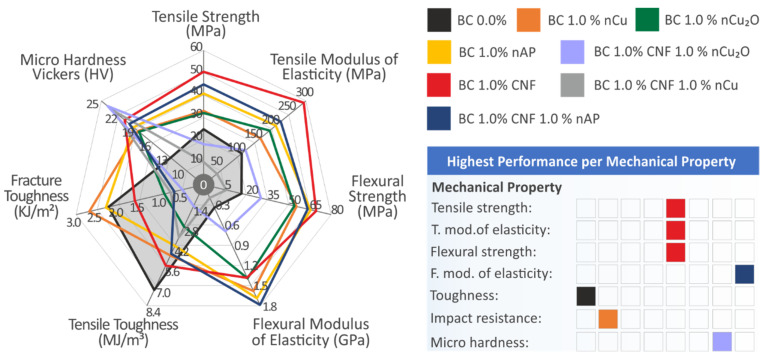
A summary of the mechanical performance results for all tested materials (**left**) and a table for the materials with the highest measured/calculated values (**right**) are shown. The shaded region on the spider graph represents the pure resin’s measured mechanical characteristics.

All fillers had a positive effect on the mechanical response of the matrix material, with the Biomed Clear 1 wt.% CNF showing the greatest enhancement. In the case of binary nano inclusions, this effect was reduced in most tests, or degraded performance when compared to the matrix material was reported. It should be mentioned that a low filler loading of 1 wt.% was selected for all cases, while the nanocomposites with two nanofillers had double the filler concentration in the matrix material, which probably led to saturation phenomena in the matrix material. Mechanical test results are summarized in Figure 15. The highest measured values for each test are indicated in Figure 15, while the shaded region on the spider graph represents the pure resin’s measured mechanical characteristics. The thermal stability of the material was not affected by the addition of the fillers in all cases, while the morphology analysis showed that the dispersion of the NPs in the matrix was fine with the methodology followed, with no visible agglomerations observed for most of the cases studied. Regarding the antibacterial performance, copper-based and AP fillers introduced antibacterial performance in the matrix material and verified their antibacterial properties in the fabricated nanocomposites.

The nanocomposite materials fabricated herein with Biomed Clear vat photopolymerization resin as the matrix material have the potential to be used in medical applications requiring enhanced mechanical performance, especially since the cost increase for the preparation of these nanocomposite materials is not significant when compared to the matrix material. There is no need for special equipment in the shear mixing methodology followed in this work, and adding the filler raises the material cost by about 1.5–2.5% in all cases for the loadings studied in the work. Additional preparation costs are evaluated to be insignificant for industrial-scale applications. In the future, further research can be conducted towards the optimization of the process and the development of materials meeting specific mechanical or medicinal criteria.

## Figures and Tables

**Table 1:** Raman spectra.

Wavenumber (cm^−1^)	Assignment
450	CCC, COC, OCC, OCO skeletal bending [70]
531–534	
585	Phenyl ring vibration [75]
690	Low-frequency vibrations of the pyranoid ring [71,72]
1343	C–C-H, C-O–H, and O-C-H [73]
1523–1524	–C=C- [74]

**Table 2:** AFM measurements.

Material	Rq (nm)	Ra (nm)	Rz (nm)
BC 0 wt.%	41.6	33.4	267.4
BC 1 wt.% CNF	176.5	145.7	1027.3
BC 1 wt.% Cu	64.7	50.7	450.8
BC 1 wt.% Cu, 1 wt.% CNF	39	28.5	386.6
BC 1 wt.% Cu_2_O	176.5	145.7	1027.3
BC 1 wt.% CNF, 1 wt.% Cu_2_O	45.6	34.9	451.9
BC 1 wt.% AP	267.5	218.9	1579.9
BC 1 wt.% CNF, 1 wt.% AP	145.3	115.3	1037.2

**Table 3:** Toughness calculated (MJ/m^3^) and deviation for all materials, Impact strength (kJ/m^2^) and deviation for all materials, and Vickers-microhardness (HV) and deviation for all materials.

Material	Tensile Toughness (MJ/m^3^) (Deviation)	Impact Toughness (MJ/m^3^) (Deviation)	Microhardness (HV)
BC 0 wt.%	9.4 (1.0)	2.7 (0.2)	14.5 (0.8)
BC 1 wt.% CNF	6.4 (0.4)	1.9 (0.1)	22.9 (0.8)
BC 1 wt.% Cu	6.6 (0.8)	3.1 (0.2)	20.6 (1.0)
BC 1 wt.% Cu, 1 wt.% CNF	4.1 (0.3)	1.0 (0.1)	24.8 (0.9)
BC 1 wt.% Cu_2_O	3.4 (0.2)	1.3 (0.2)	19.9 (0.7)
BC 1 wt.% CNF, 1 wt.% Cu_2_O	1.9 (0.2)	0.6 (0.03)	25.6 (0.8)
BC 1 wt.% AP	4.6 (0.2)	2.4 (0.03)	21.8 (1.2)
BC 1 wt.% CNF, 1 wt.% AP	6.3 (0.5)	1.1 (0.2)	22.3 (1.0)

## Data Availability

The data presented in this study are available upon request from the corresponding author.

## Supplementary Materials

The following supporting information can be downloaded at: https://www.mdpi.com/article/10.3390/polym14091903/s1, Figure S1: Graphs of the DSC analysis: (A) Biomed Clear pure resin, (B) Biomed Clear with 1 wt.% CNF, (C) Biomed Clear with 1 wt.% Cu, (D) Biomed Clear with 1 wt.% CNF and 1 wt.% Cu, (E) Biomed Clear with 1 wt.% Cu_2_O, (F) Biomed Clear with 1 wt.% CNF and 1 wt.% Cu_2_O, (G) Biomed Clear with 1 wt.% Antibacterial Powder, (H) Biomed Clear with 1 wt.% CNF and 1 wt.% Antibacterial Powder. Arrows indicate a phase change in the material. Figure S2: AFM measurements: (A) AFM setup employed in this work, (B) Biomed Clear pure resin; (C) Biomed Clear 1 wt.% CNF, (D) Biomed Clear 1.0 wt.% Cu, (E) Biomed Clear 1 wt.% CNF and 1 wt.% Cu, (F) Biomed Clear 1 wt.% Cu_2_O (G) Biomed Clear 1 wt.% CNF and 1 wt.% Cu_2_O, (H) Biomed Clear 1 wt.% Antibacterial Powder, (I) Biomed Clear 1 wt.% CNF and 1 wt.% Antibacterial Powder. Figure S3: (A) Toughness calculated (MJ/m3) and deviation for all materials (B) Impact strength (kJ/m2) and deviation for all materials (C) Vickers-microhardness (HV) and deviation for all materials. Figure S4: (A) Typical gram-negative *E. coli* morphology and captures of Petri dishes, and related inhibition zones measurements of the nanocomposites: (B) Pure Biomed Clear, (C) Biomed Clear 1 wt.% CNF, (D) Biomed Clear 1 wt.% Cu, (E) Biomed Clear 1 wt.% CNF and 1 wt.% Cu, (F) Biomed Clear 1 wt.% Cu_2_O, (G) Biomed Clear 1 wt.% CNF and 1 wt.% Cu_2_O, (H) Biomed Clear 1 wt.% Antibacterial Powder, (I) Biomed Clear 1 wt.% CNF and 1 wt.% Antibacterial Powder. Figure S5: (A) Typical gram-positive S. Aureus morphology and captures of Petri dishes, and related inhibition zones measurements of the nanocomposites: (B) Pure Biomed Clear, (C) Biomed Clear 1 wt.% CNF, (D) Biomed Clear 1 wt.% Cu, (E) Biomed Clear 1 wt.% CNF and 1 wt.% Cu, (F) Biomed Clear 1 wt.% Cu_2_O, (G) Biomed Clear 1 wt.% CNF and 1 wt.% Cu_2_O, (H) Biomed Clear 1 wt.% Antibacterial Powder, (I) Biomed Clear 1 wt.% CNF and 1 wt.% Antibacterial Powder.
