# Supplementary material for: Multi-Functional 3D-Printed Vat Photopolymerization Biomedical-Grade Resin Reinforced with Binary Nano Inclusions: The Effect of Cellulose Nanofibers and Antimicrobial Nanoparticle Agents
Source: Polymers (Basel). 2022 May 6;14(9):1903. doi: 10.3390/polym14091903 (PMC9100280; doi:10.3390/polym14091903)
Supplement: Supplementary file 1 [file polymers-14-01903-s001.zip › polymers-1700402-supplementary.pdf]

Supplementary data for article:

Multi-Functional 3D-Printed Vat Photopolymerization Biomedical-Grade Resin Reinforced with Binary Nano Inclusions: The Effect of Cellulose Nanofibers and Antimicrobial Nanoparticle Agents

Nectarios Vidakis [1], Markos Petousis [1,*], Nikolaos Michailidis [2,3], Vassilis Papadakis [4], Apostolos Korlos [5], Nikolaos Mountakis [1] and Apostolos Argyros [2,3]

1 Mechanical Engineering Department, Hellenic Mediterranean University, Estavromenos, 71410 Heraklion, Greece; vidakis@hmu.gr (N.V.); mountakis@hmu.gr (N.M.)
2 Physical Metallurgy Laboratory, Mechanical Engineering Department, School of Engineering, Aristotle University of Thessaloniki, 54124 Thessaloniki, Greece; nmichail@auth.gr (N.M.); aposargy1@ee.duth.gr (A.A.)
3 Centre for Research & Development of Advanced Materials (CERDAM), Center for Interdisciplinary Research and Innovation, Balkan Centre, Building B’, 10th km Thessaloniki-Thermi Road, 57001 Thessaloniki, Greece
4 Institute of Molecular Biology and Biotechnology, Foundation for Research and Technology—Hellas, P.O. Box 1527, 70013 Heraklion, Greece; vassilis_papadakis@imbb.forth.gr
5 Department of Industrial Engineering and Management, International Hellenic University, 14th km Thessaloniki—N. Moudania, Thermi, 57001 Thessaloniki, Greece; apkorlos@ihu.gr
* Correspondence: markospetousis@hmu.gr; Tel.: +30-2810379227

Abstract: This study introduced binary nanoparticle (NP) inclusions into a biomedical-grade photosensitive resin (Biomed Clear-BC). Multi-functional three-dimensional (3D) printed objects were manufactured via vat photopolymerization additive manufacturing (AM) technique. Cellulose nanofibers (CNFs) as one dimensional (1D) nanomaterial have been utilized for the mechanical reinforcement of the resin, while three different spherical NPs; namely copper NPs (nCu), copper oxide NPs (nCuO), and a commercial antimicrobial powder (nAP) endowed the antimicrobial character. The nanoparticle loading was kept constant at 1.0 wt.% to elucidate any synergistic effects as a function of the filler loading. Raman, thermogravimetric analysis (TGA), and differential scanning calorimetry (DSC) revealed the chemical/spectroscopic and thermal properties of the different manufactured samples. Scanning electron microscopy and Atomic Force Microscopy (AFM) revealed the morphology of the samples. Mechanical properties revealed the reinforcement mechanisms. Namely, BC/CNF (1.0 wt.%) exhibited 102% and 154% enhancement in strength and modulus, while BC/CNF(1.0wt.%)/AP(1.0 wt.%) 95% and 101%, as well as an antibacterial property, which was studied using a screening agar well diffusion method. This study opens the route towards novel multi-functional materials for vat photopolymerization 3D printing biomedical applications, where mechanical reinforcement and antibacterial performance are typically required in the operational environment.

Figure S1. DSC graphs for all the materials tested

Exothermic and endothermic graphs acquired during the DSC analysis implemented within the context of this work, for all the materials tested. The phase changes are indicated in the graphs. Please see the manuscript, for analysis and discussion of the DSC curves.

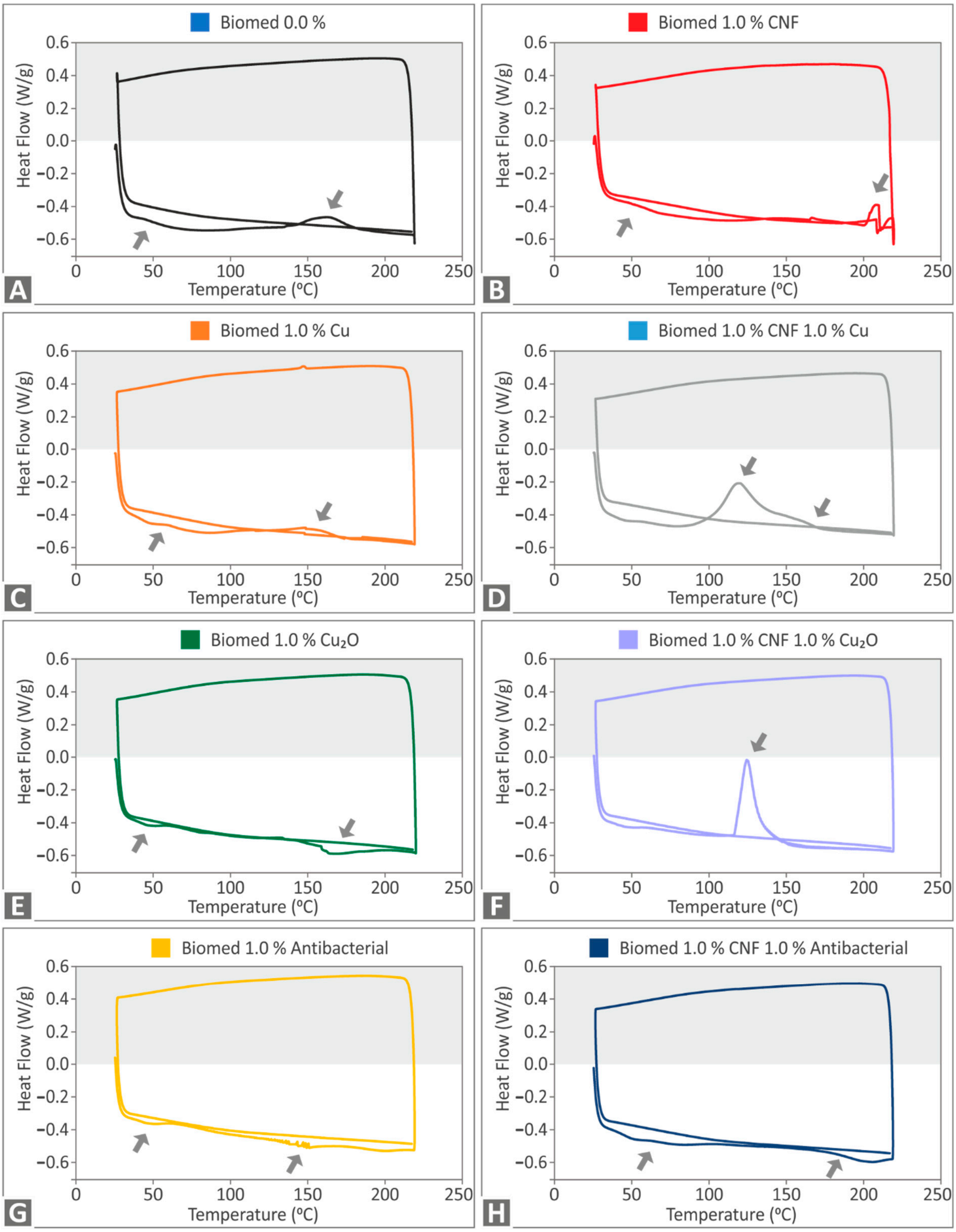

Figure S1. Graphs of the DSC analysis: (A) Biomed Clear pure resin, (B) Biomed Clear with 1 wt.% CNF, (C) Biomed Clear with 1 wt.% Cu, (D) Biomed Clear with 1 wt.% CNF and 1 wt.% Cu, (E) Biomed Clear with 1 wt.% Cu_2O, (F) Biomed Clear with 1 wt.% CNF and 1 wt.% Cu_2O, (G) Biomed Clear with 1 wt.% Antibacterial

Powder, (H) Biomed Clear with 1 wt.% CNF and 1 wt.% Antibacterial Powder. Arrows indicate a phase change in the material.

Figure S2. AFM surface morphology of the filament, produced for all the materials tested

Filament side surface morphology, as it was acquired within the context of this work, for all the materials tested. The calculated surface roughness values in each case are also indicated. Please see the manuscript, for analysis and discussion of the AFM measurements.

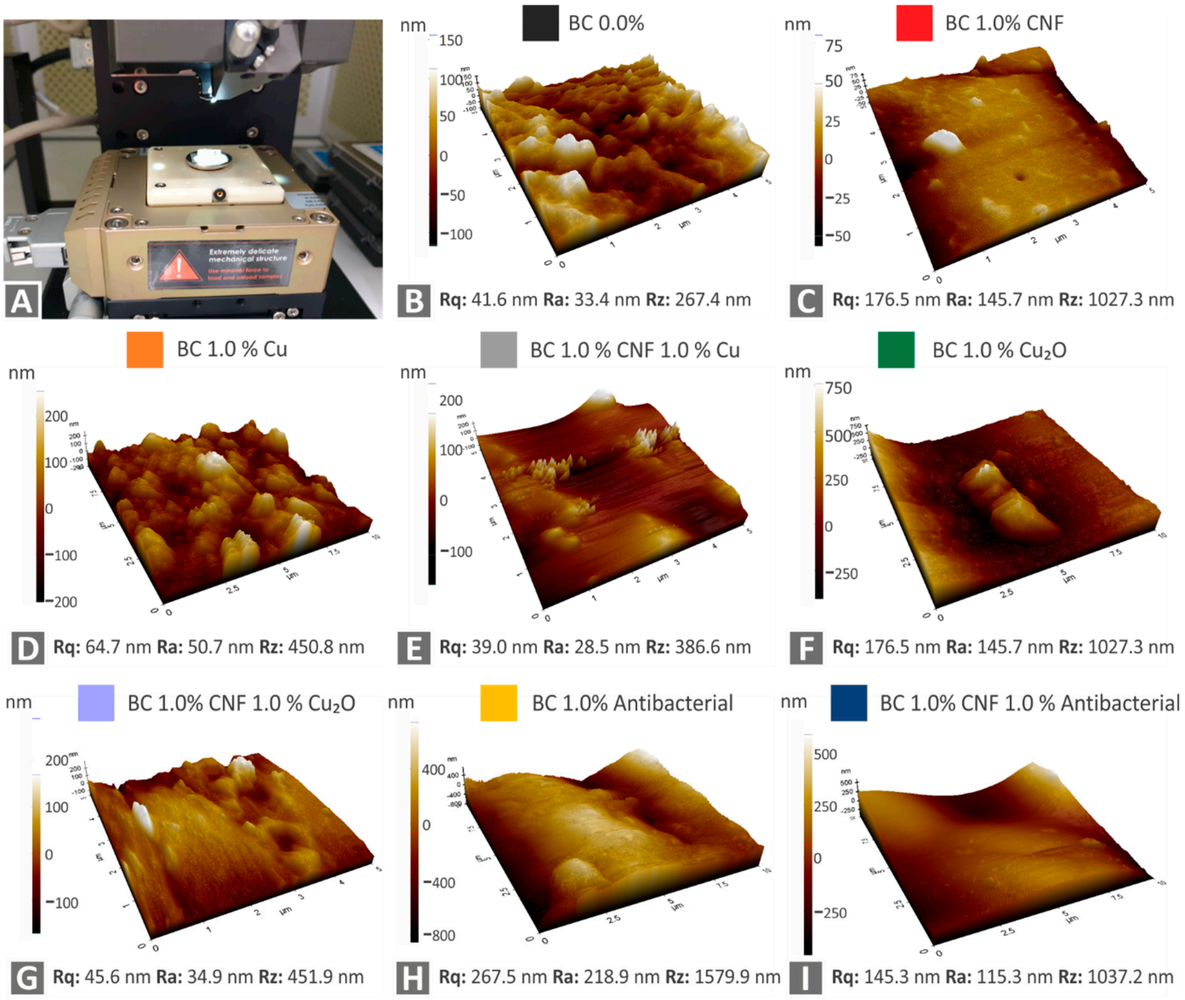

Figure S2. AFM measurements: (A) AFM setup employed in this work, (B) Biomed Clear pure resin; (C) Biomed Clear 1 wt.% CNF, (D) Biomed Clear 1.0 wt.% Cu, (E) Biomed Clear 1 wt.% CNF and 1 wt.% Cu, (F) Biomed Clear 1 wt.% Cu_2O (G) Biomed Clear 1 wt.% CNF and 1 wt.% Cu_2O, (H) Biomed Clear 1 wt.% Antibacterial Powder, (I) Biomed Clear 1 wt.% CNF and 1 wt.% Antibacterial Powder.

Figure S3. Tensile toughness values, Impact toughness values and Microhardness measurements, with their deviations, for all the materials tested

(A) Tensile toughness values and their deviation, calculated within the context of this work, for all the materials tested. The tensile toughness is calculated as the integral of the stress vs strain graph and is an indicator of the absorbed energy of the material, during the mechanical test.
(B) Impact toughness values and their deviation, calculated within the context of this work, for all the materials tested, from the corresponding experimental procedure (Charpy's impact test on notched specimens)
(C) Vickers microhardness values and their deviation, measured within the context of this work, for all the materials tested.

Please see the manuscript, for analysis and discussion of the data presented in the charts.

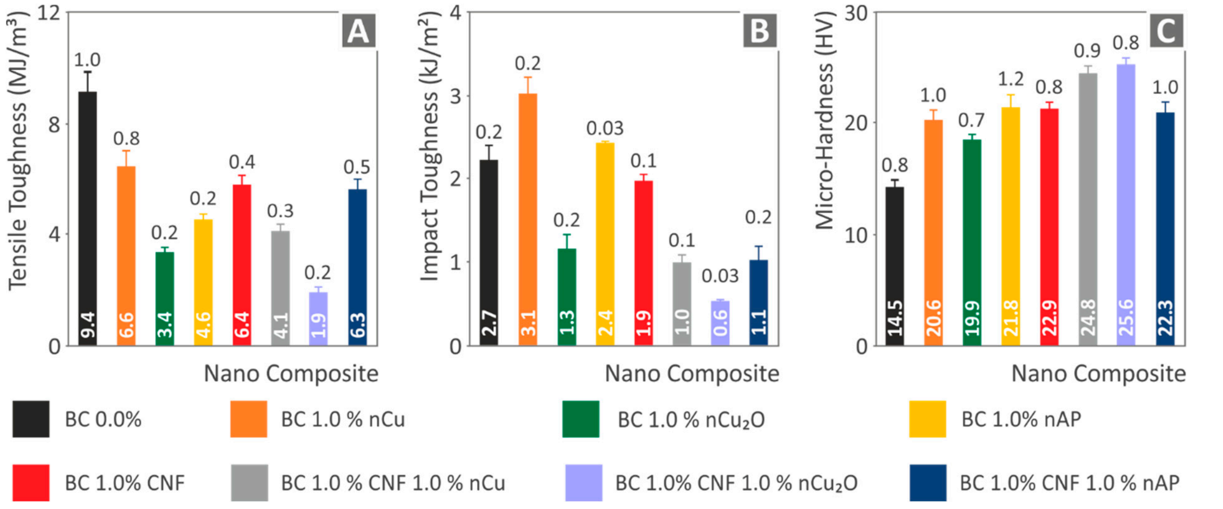

Figure S3. (A) Toughness calculated (MJ/m^3) and deviation for all materials (B) Impact strength (kJ/m^2) and deviation for all materials (C) Vickers-microhardness (HV) and deviation for all materials.

Figure S4. E. Coli Inhibition zones, for all the materials tested

The E. Coli bacterium captured in the optical microscope and the developed IZs, of all the materials tested, during the agar well diffusion screening process, within the context of this work, showing the performance of the materials against the bacterium growth. Please see the manuscript, for further analysis and discussion.

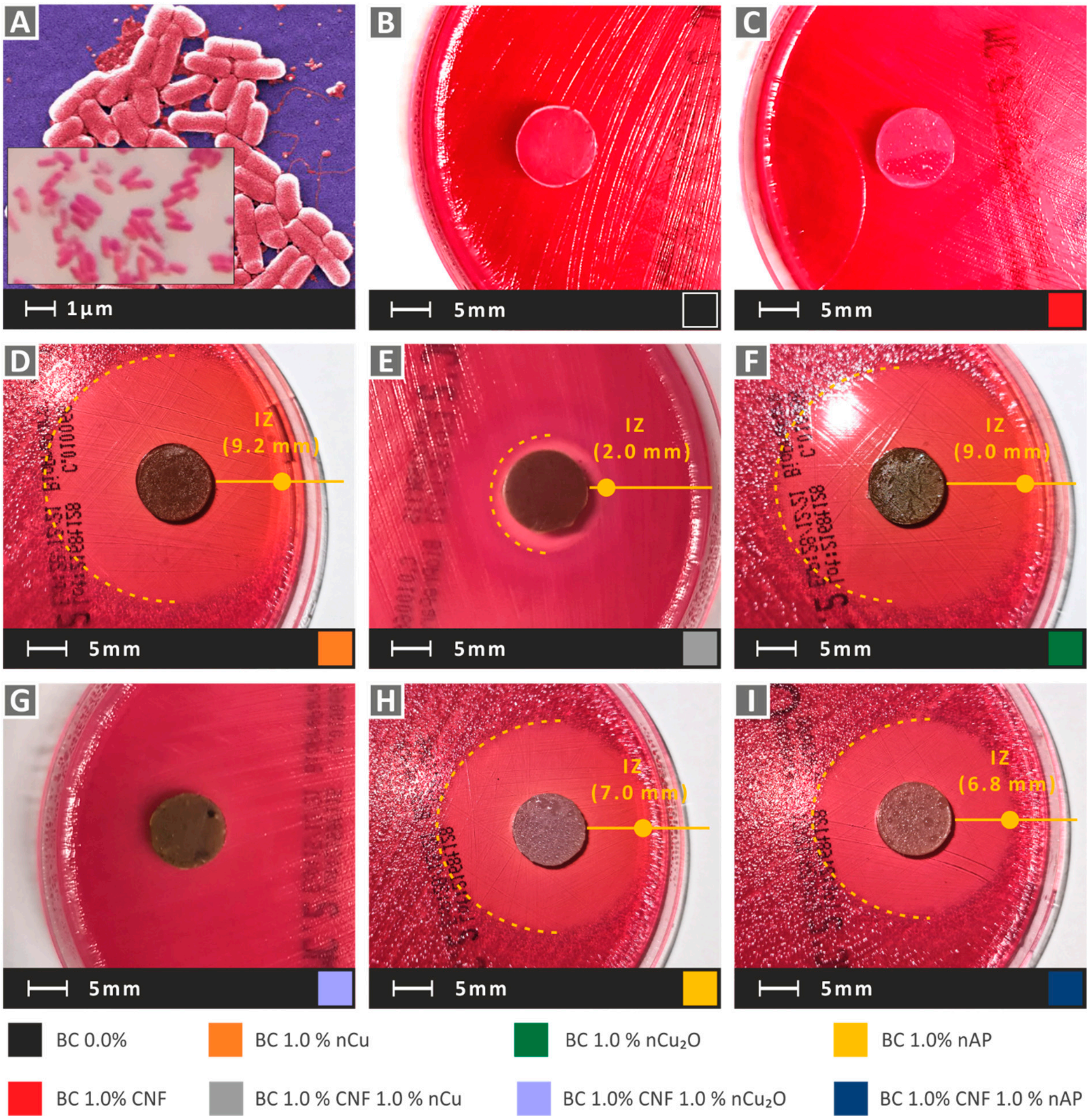

Figure S4. (A) Typical gram-negative *E. Coli* morphology and captures of Petri dishes, and related inhibition zones measurements of the nanocomposites: (B) Pure Biomed Clear, (C) Biomed Clear 1 wt.% CNF, (D) Biomed Clear 1 wt.% Cu, (E) Biomed Clear 1 wt.% CNF and 1 wt.% Cu, (F) Biomed Clear 1 wt.% Cu_2O, (G) Biomed Clear 1 wt.% CNF and 1 wt.% Cu_2O, (H) Biomed Clear 1 wt.% Antibacterial Powder, (I) Biomed Clear 1 wt.% CNF and 1 wt.% Antibacterial Powder.

Figure S5. S. Aureus Inhibition zones, for all the materials tested

The S. Aureus bacterium captured in the optical microscope and the developed IZs, of all the materials tested, during the agar well diffusion screening process, within the context of this work, showing the performance of the materials against the bacterium growth. Please see the manuscript, for further analysis and discussion.

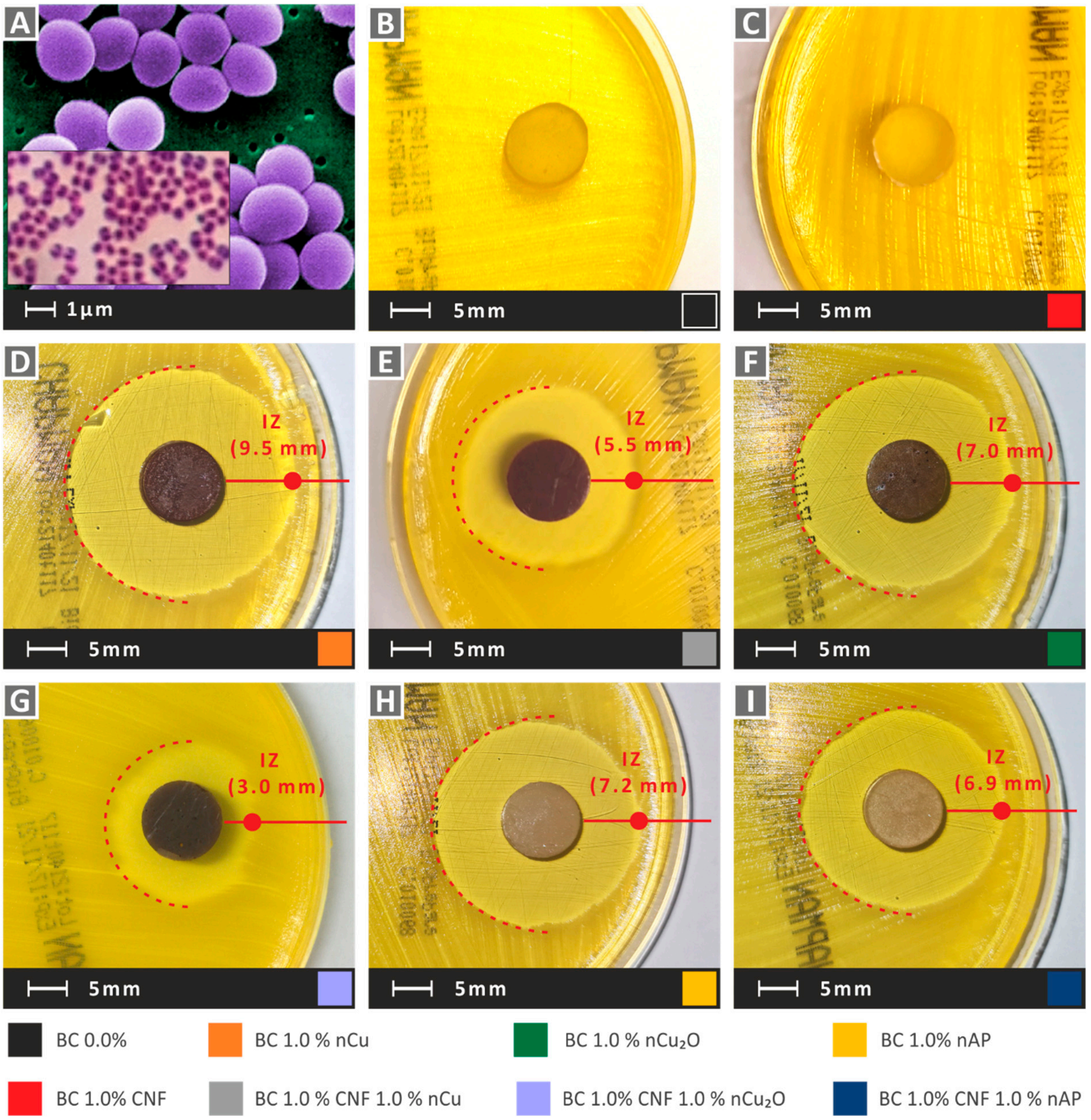

Figure S5. (A) Typical gram-positive *S. Aureus* morphology and captures of Petri dishes, and related inhibition zones measurements of the nanocomposites: (B) Pure Biomed Clear, (C) Biomed Clear 1 wt.% CNF, (D) Biomed Clear 1 wt.% Cu, (E) Biomed Clear 1 wt.% CNF and 1 wt.% Cu, (F) Biomed Clear 1 wt.% Cu_2O, (G) Biomed Clear 1 wt.% CNF and 1 wt.% Cu_2O, (H) Biomed Clear 1 wt.% Antibacterial Powder, (I) Biomed Clear 1 wt.% CNF and 1 wt.% Antibacterial Powder.
